# Supplementary material for: Genetic Characterization of Rat Hepatic Stellate Cell Line PAV-1
Source: Cells. 2023 Jun 11;12(12):1603. doi: 10.3390/cells12121603 (PMC10297474; doi:10.3390/cells12121603)
Supplement: Supplementary file 1 [file cells-12-01603-s001.zip › Figure S1,S3, and S4.pdf]

cells MDPI

Supplements

Genetic Characterization of Rat Hepatic Stellate Cell Line PAV-1

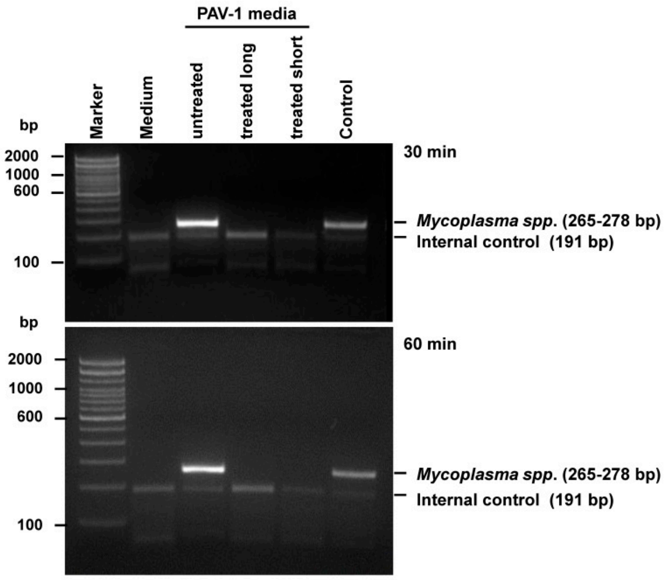

Figure S1. Testing for *Mycoplasma spp.* infection in PAV-1 cells. In the depicted analysis, normal control medium (2 µl) and conditioned PAV-1 medium (2 µl) before and after treatment with the Plasmocin®-Mycoplasma elimination reagent were tested for *Mycoplasma* infection using the Venor®GeM OneStep kit. As a positive control, 2 µl of the positive control included in the kit system was co-amplified. In this assay, the internal control in each sample giving rise to a distinct 191-bp fragment indicates a successfully performed PCR, while an amplicon of 265-278 bp in size indicates a contamination with *Mycoplasma spp*. The PCR products were separated on a 2% standard agarose gel including ethidium bromide in TAE buffer (90 V) and amplicons visualized after 30 or 60 min using a standard gel imager.

Figure S2. Short tandem repeat (STR) profiling of AML12 cells. Genomic DNA from AML12 was profiled with the CellCheck™ Mouse STR system that contains 19 species specific consensus STR markers proposed by the Consortium for Mouse Cell Line Authentication for STR profiling in mouse. The profile of the 19 markers suggests a unique STR profile. See separate file.

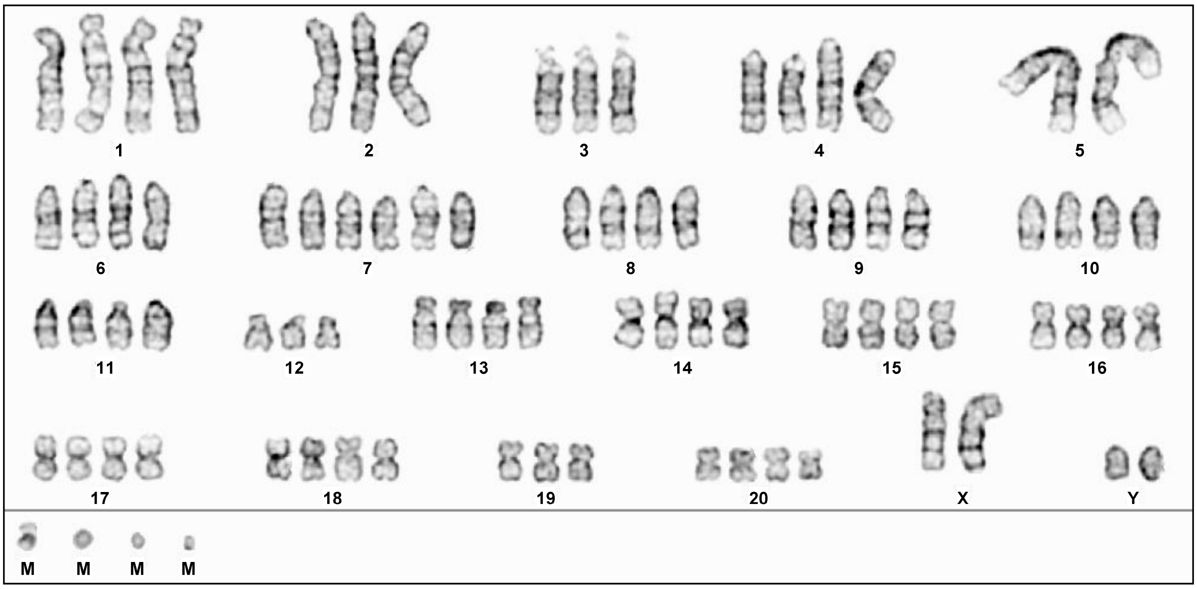

Figure S3. GTG-banded karyotype of a tetraploid PAV-1 cell. Most of the autosomes except RNO7 and RNO12 are in four copies.

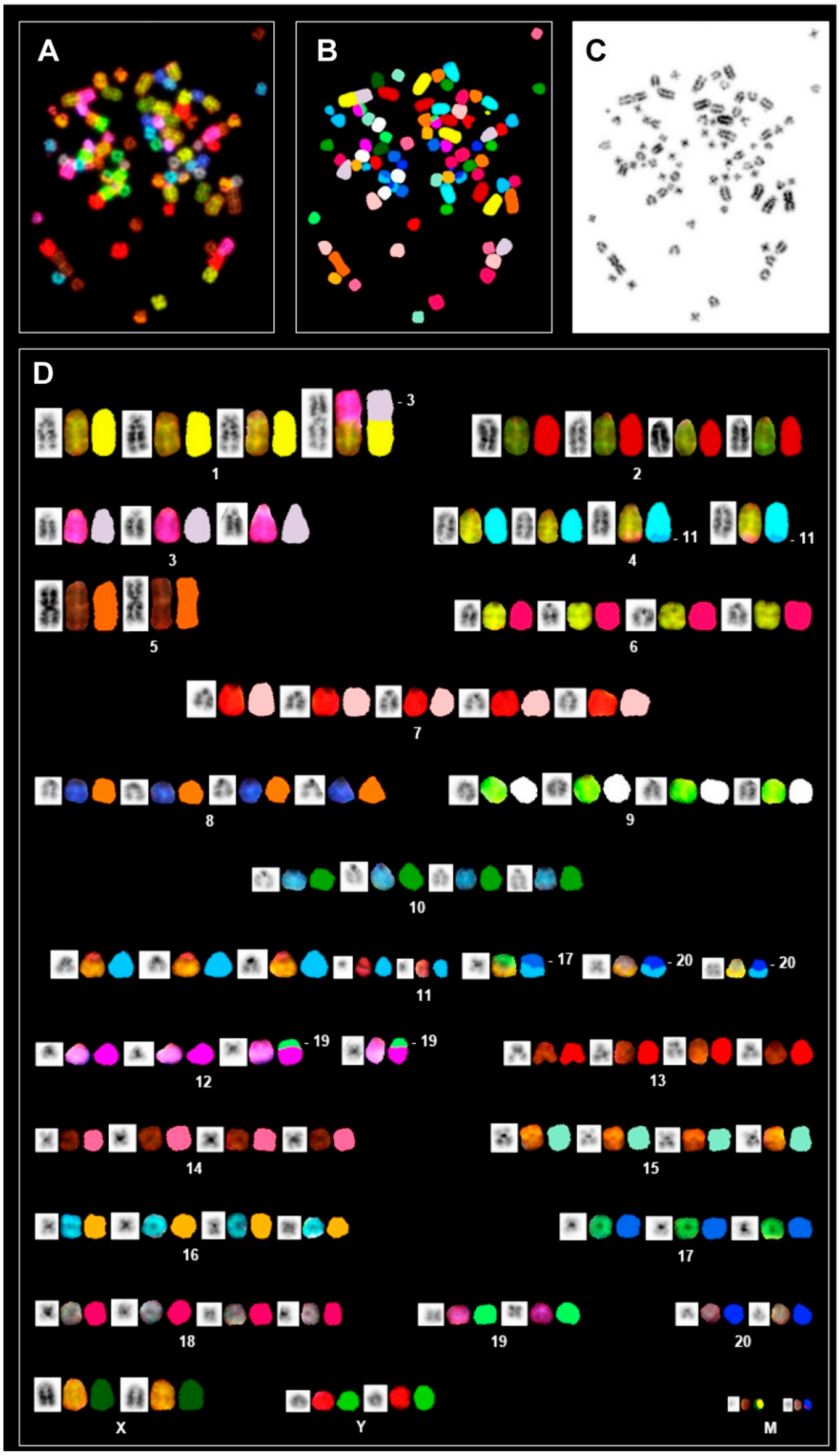

Figure S4. Spectral karyotyping of a tetraploid PAV-1 cell line metaphase. (**A**) RGB image after hybridization of metaphase with the SKY probe cocktail. (**B**) Classified pseudo-color image of the same hybridized metaphase. (**C**) Inverted DAPI-stained image. (**D**) Karyotype of the hybridized metaphase showing spectrally classified, pseudo-colored chromosomes (*right*) compared with its inverted DAPI-stained chromosomes (*left*) and corresponding RGB image (*middle*). Chromosomes involved in rearrangements display two different color codes. Note the additional sporadic translocations, t(1;3) and t(11;17) in karyotype.

Figure S5. Short tandem repeat (STR) profiling in PAV-1 cells**.** Depicted are the electropherograms of the 19 species specific markers used for cell authentication in the Multiplex Cell Authentication (MCA) assay. For this analysis, genomic DNA from PAV-1 cells was isolated and the genetic profile determined using the consensus mouse STR markers 1–1, 1–2, 2–1, 3–2, 4–2, 5–5, 6–4, 6–7, 7–1, 8–1, 9–2, 11–2, 12–1, 13–1, 15–3, 17–2, 18–3, 19–2, and X-1, respectively. See separate file.
