# Supplementary material for: Genetic Characterization of Rat Hepatic Stellate Cell Line PAV-1
Source: Cells. 2023 Jun 11;12(12):1603. doi: 10.3390/cells12121603 (PMC10297474; doi:10.3390/cells12121603)
Supplement: Supplementary file 1 [file cells-12-01603-s001.zip › Figure S2.pdf]

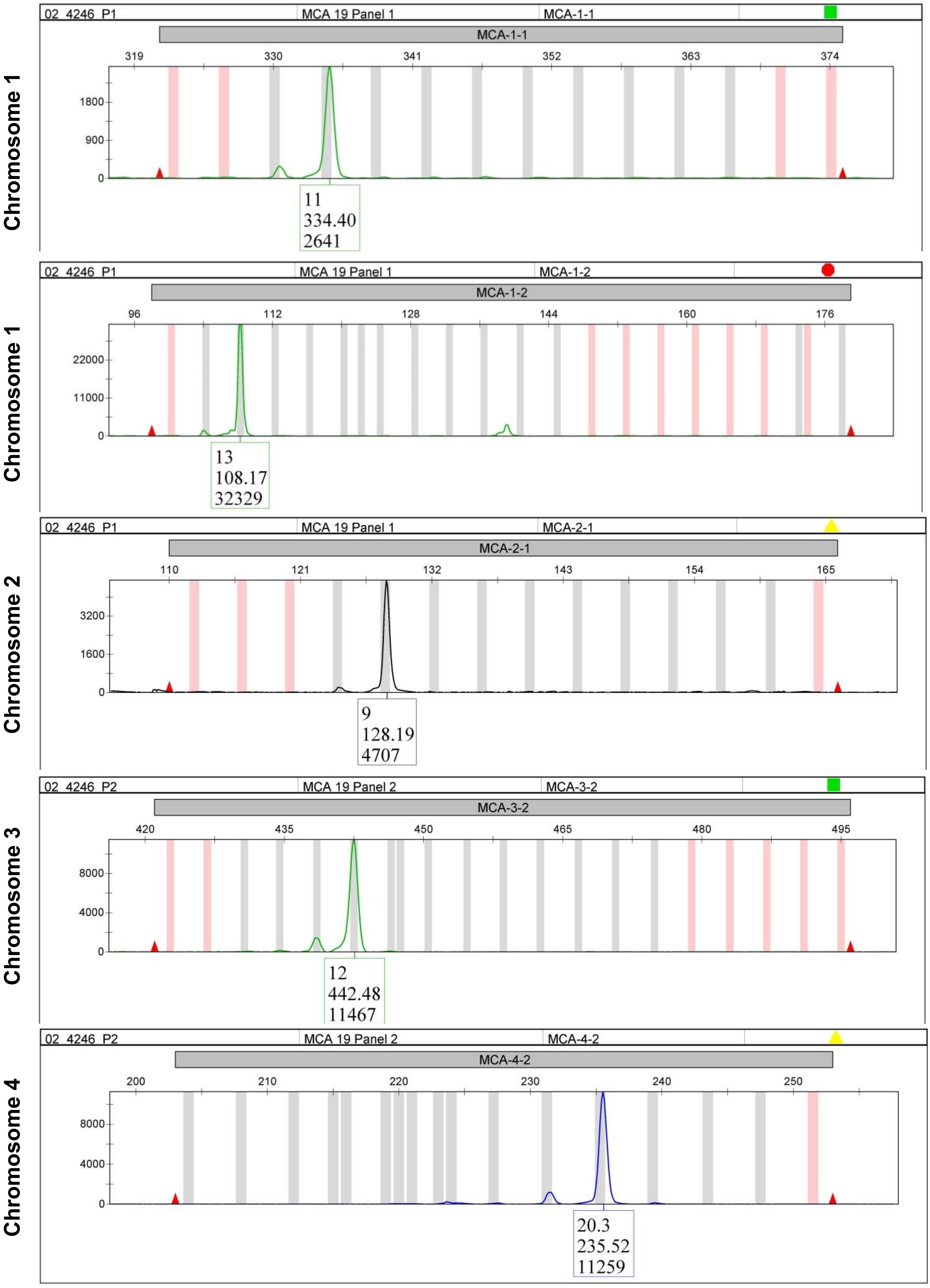

Chromosome 1
02 4246 P1
MCA 19 Panel 1
MCA-1-1
319 330 341 352 363 374
1800
900
0
11
334.40
2641
Chromosome 1
02 4246 P1
MCA 19 Panel 1
MCA-1-2
96 112 128 144 160 176
22000
11000
0
13
108.17
32329
Chromosome 2
02 4246 P1
MCA 19 Panel 1
MCA-2-1
110 121 132 143 154 165
3200
1600
0
9
128.19
4707
Chromosome 3
02 4246 P2
MCA 19 Panel 2
MCA-3-2
420 435 450 465 480 495
8000
4000
0
12
442.48
11467
Chromosome 4
02 4246 P2
MCA 19 Panel 2
MCA-4-2
200 210 220 230 240 250
8000
4000
0
20.3
235.52
11259

Chromosome 5

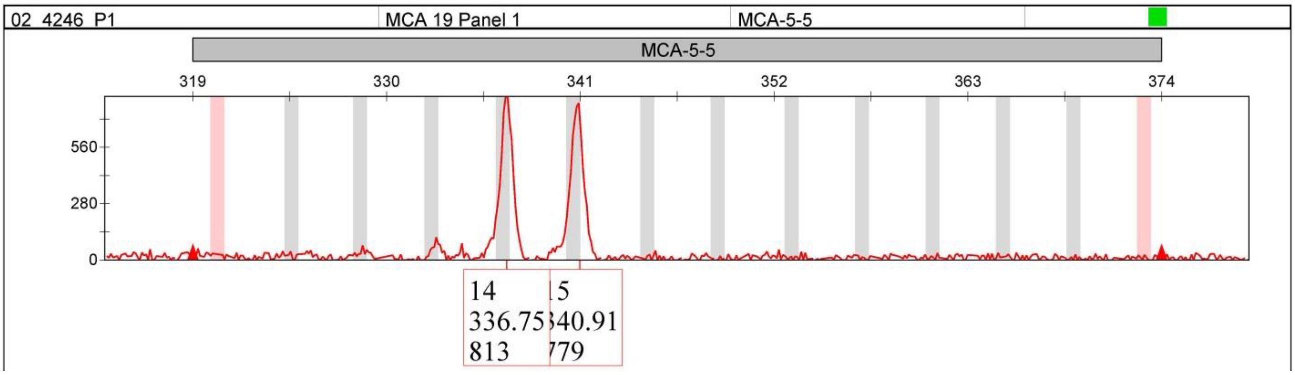

Chromosome 6

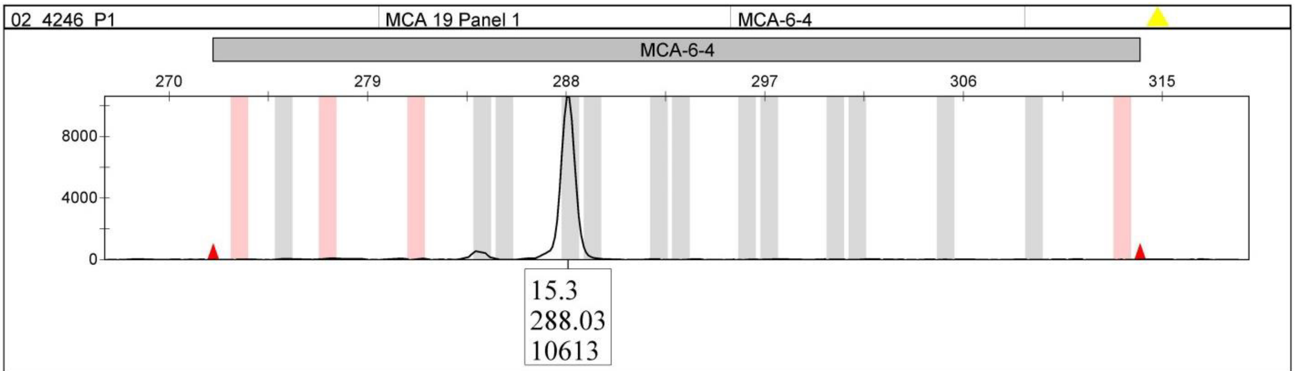

Chromosome 6

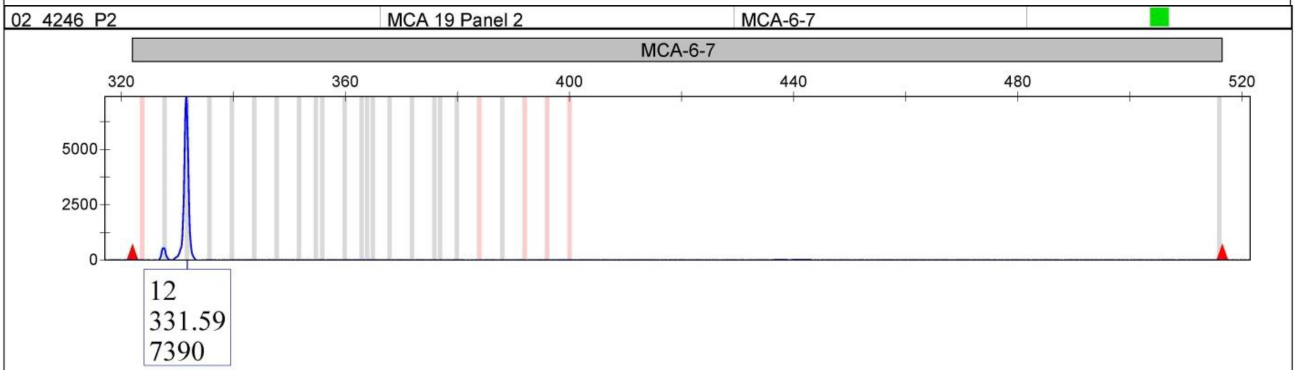

Chromosome 7

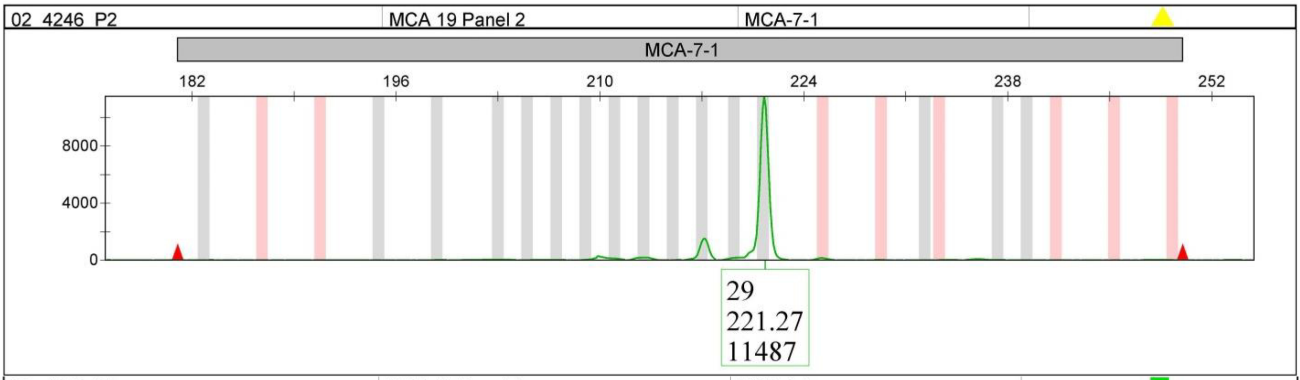

Chromosome 8

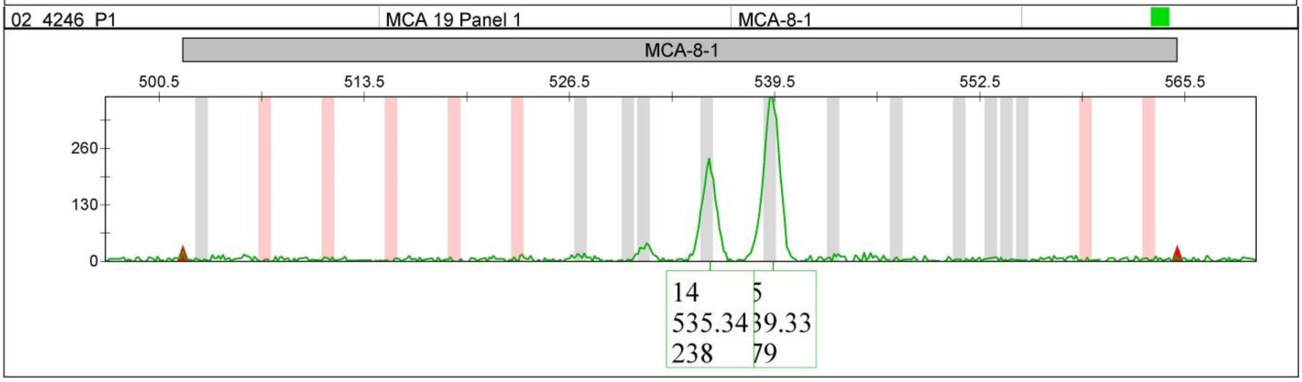

Chromosome 9

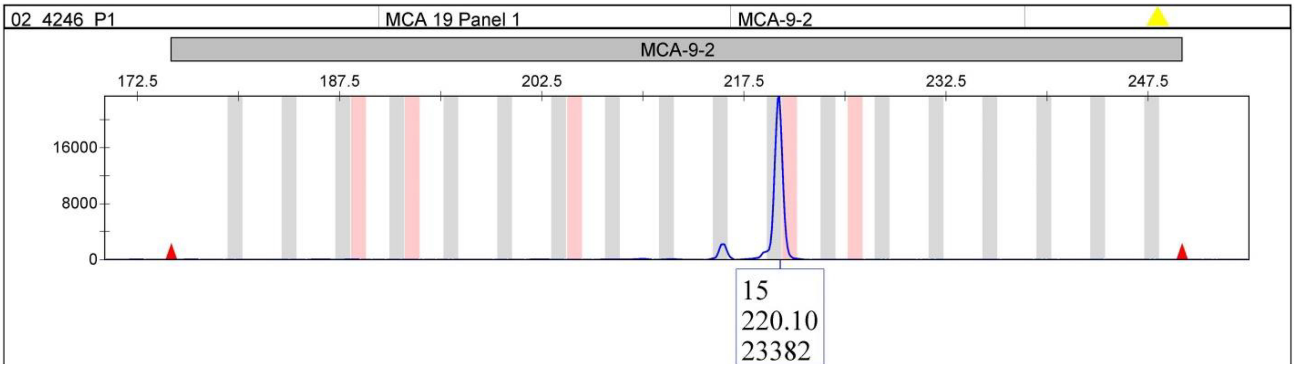

Chromosome 11

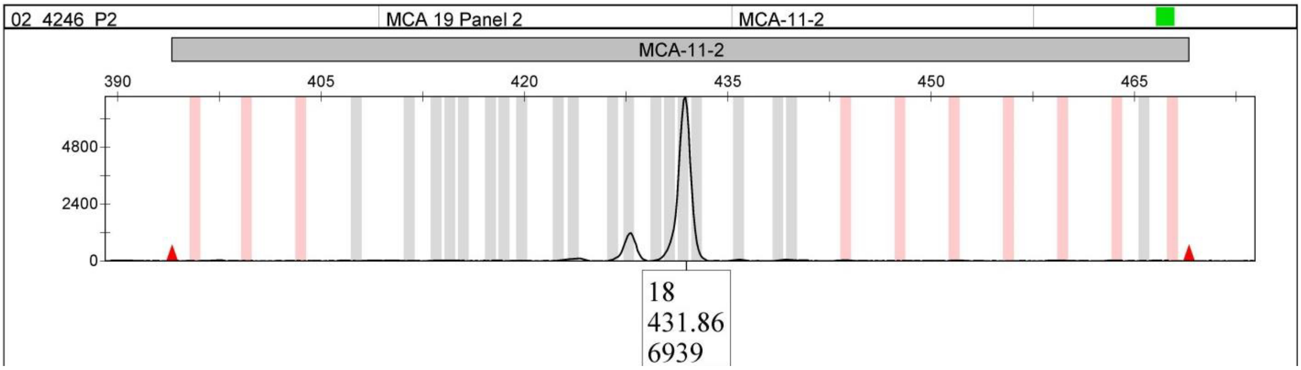

Chromosome 12

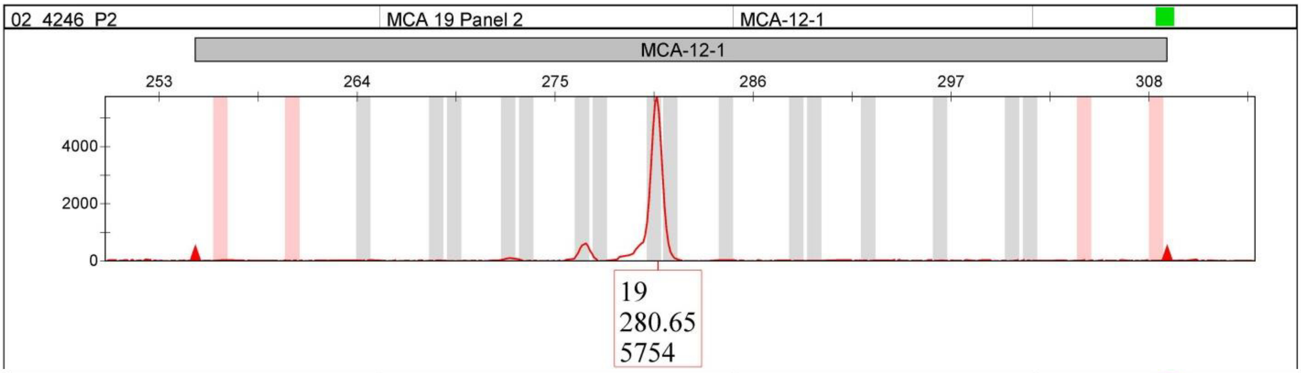

Chromosome 13

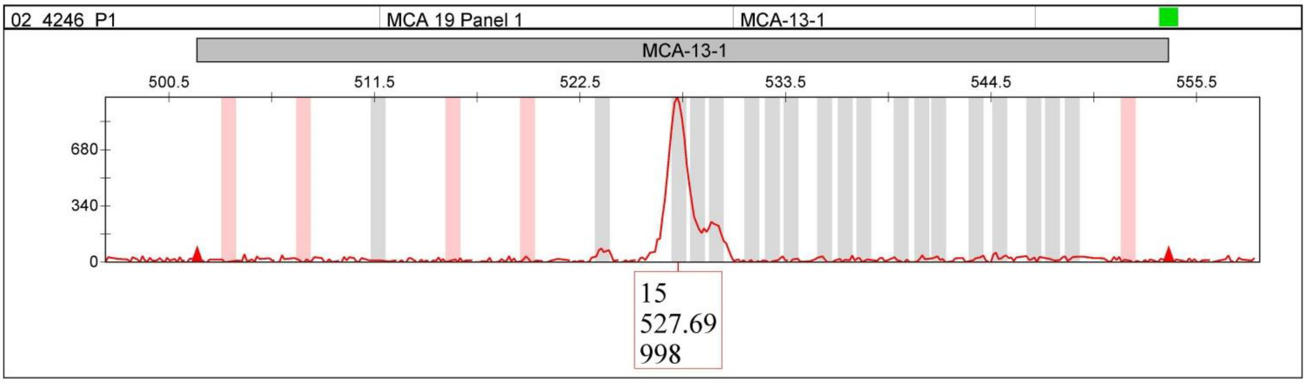

Chromosome 15

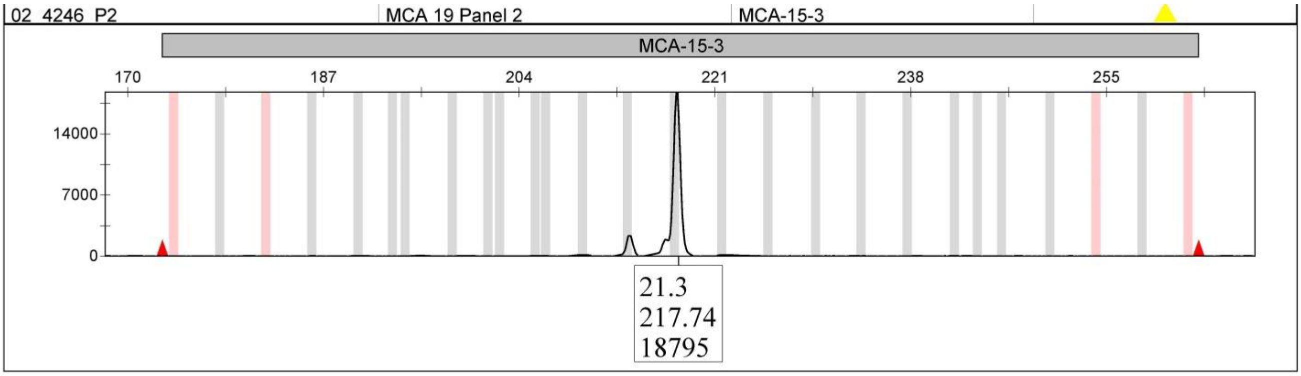

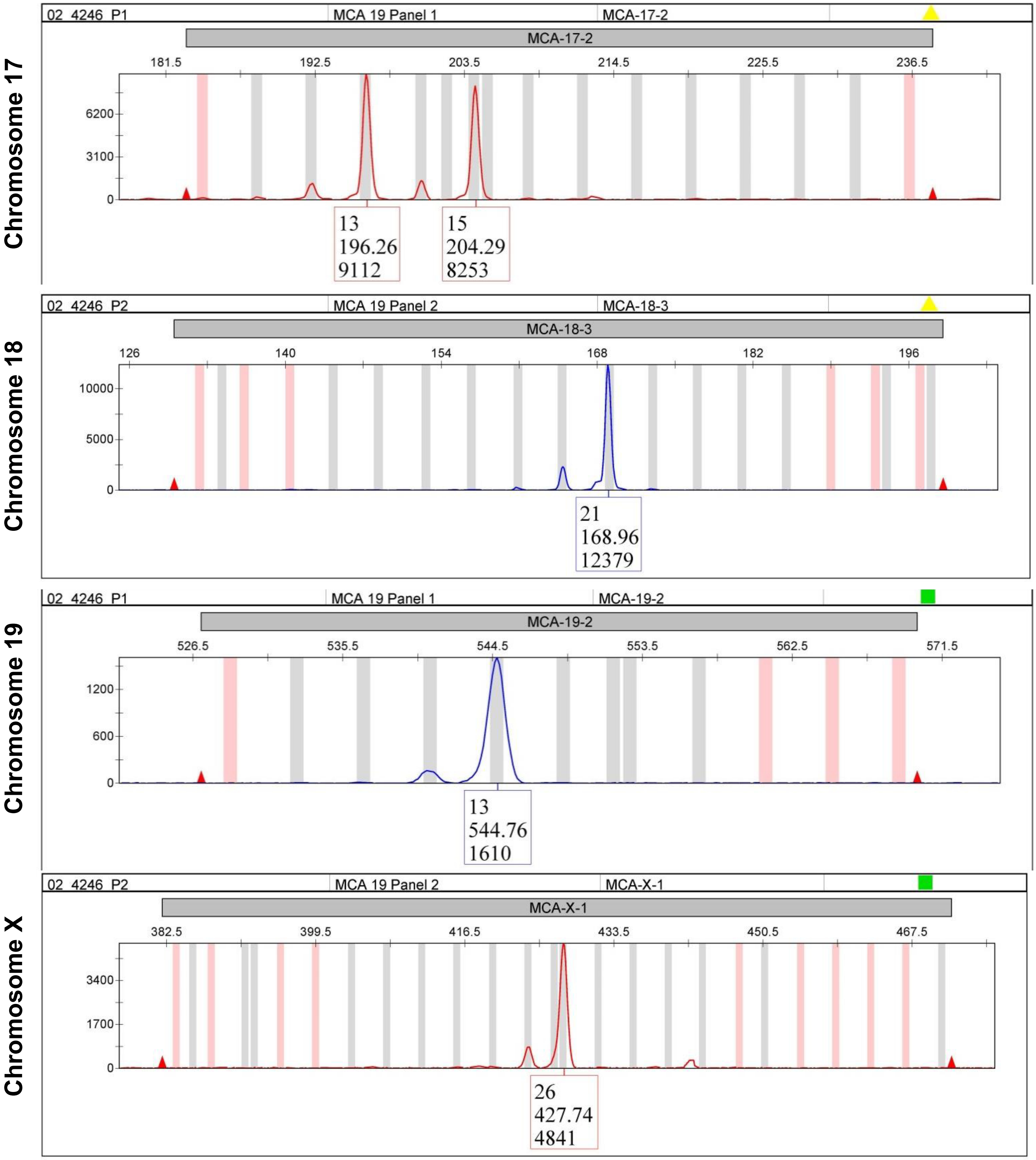
Chromosome 17
02 4246 P1
MCA 19 Panel 1
MCA-17-2
MCA-17-2
181.5
192.5
203.5
214.5
225.5
236.5
6200
3100
0
13
196.26
9112
15
204.29
8253
Chromosome 18
02 4246 P2
MCA 19 Panel 2
MCA-18-3
MCA-18-3
126
140
154
168
182
196
10000
5000
0
21
168.96
12379
Chromosome 19
02 4246 P1
MCA 19 Panel 1
MCA-19-2
MCA-19-2
526.5
535.5
544.5
553.5
562.5
571.5
1200
600
0
13
544.76
1610
Chromosome X
02 4246 P2
MCA 19 Panel 2
MCA-X-1
MCA-X-1
382.5
399.5
416.5
433.5
450.5
467.5
3400
1700
0
26
427.74
4841

AML12
