# Supplementary material for: Genetic Characterization of Rat Hepatic Stellate Cell Line PAV-1
Source: Cells. 2023 Jun 11;12(12):1603. doi: 10.3390/cells12121603 (PMC10297474; doi:10.3390/cells12121603)
Supplement: Supplementary file 1 [file cells-12-01603-s001.zip › Figure S5.pdf]

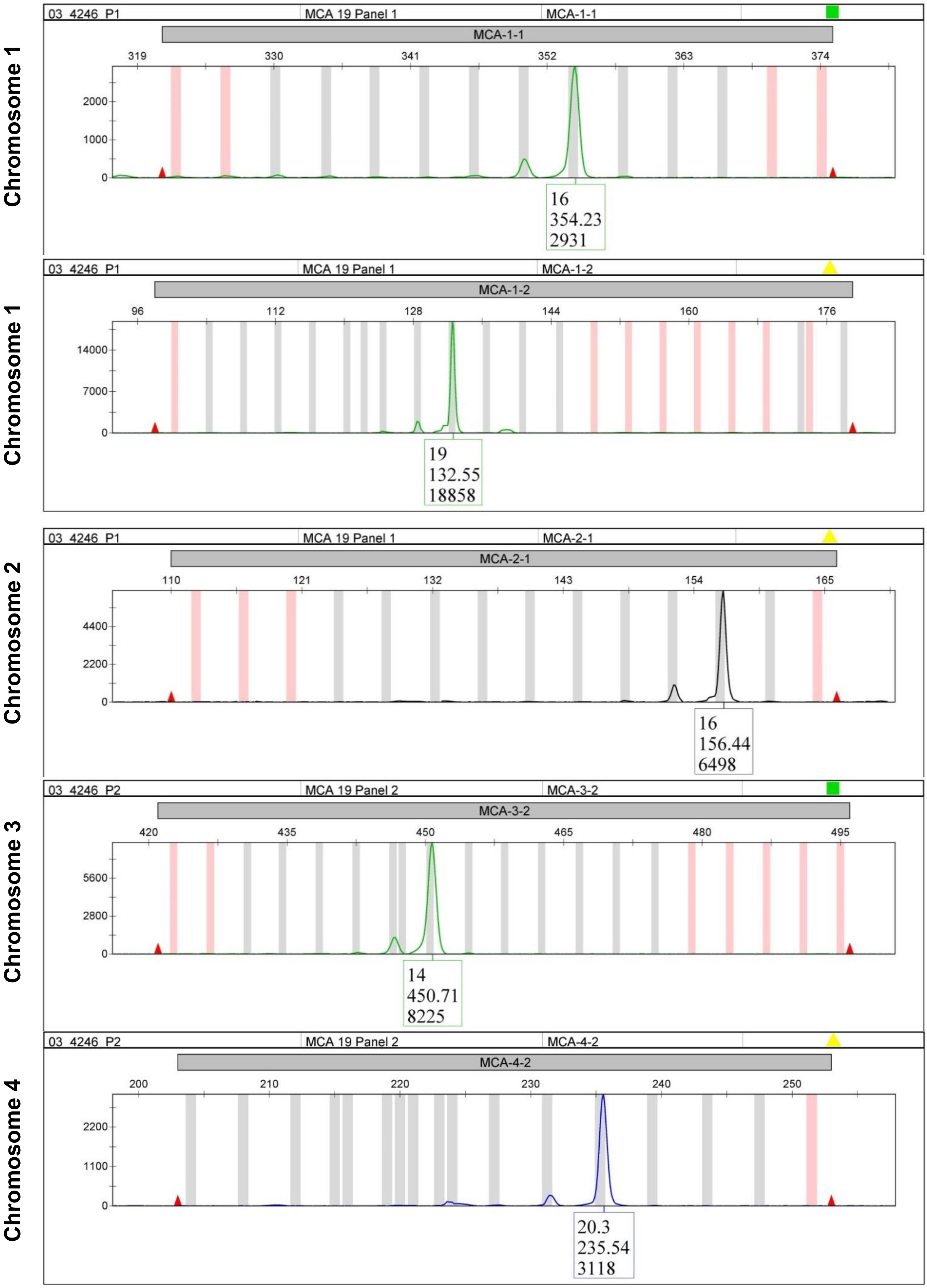
Chromosome 1
03_4246_P1
MCA 19 Panel 1
MCA-1-1
319
330
341
352
363
374
2000
1000
0
16
354.23
2931
Chromosome 1
03_4246_P1
MCA 19 Panel 1
MCA-1-2
96
112
128
144
160
176
14000
7000
0
19
132.55
18858
Chromosome 2
03_4246_P1
MCA 19 Panel 1
MCA-2-1
110
121
132
143
154
165
4400
2200
0
16
156.44
6498
Chromosome 3
03_4246_P2
MCA 19 Panel 2
MCA-3-2
420
435
450
465
480
495
5600
2800
0
14
450.71
8225
Chromosome 4
03_4246_P2
MCA 19 Panel 2
MCA-4-2
200
210
220
230
240
250
2200
1100
0
20.3
235.54
3118

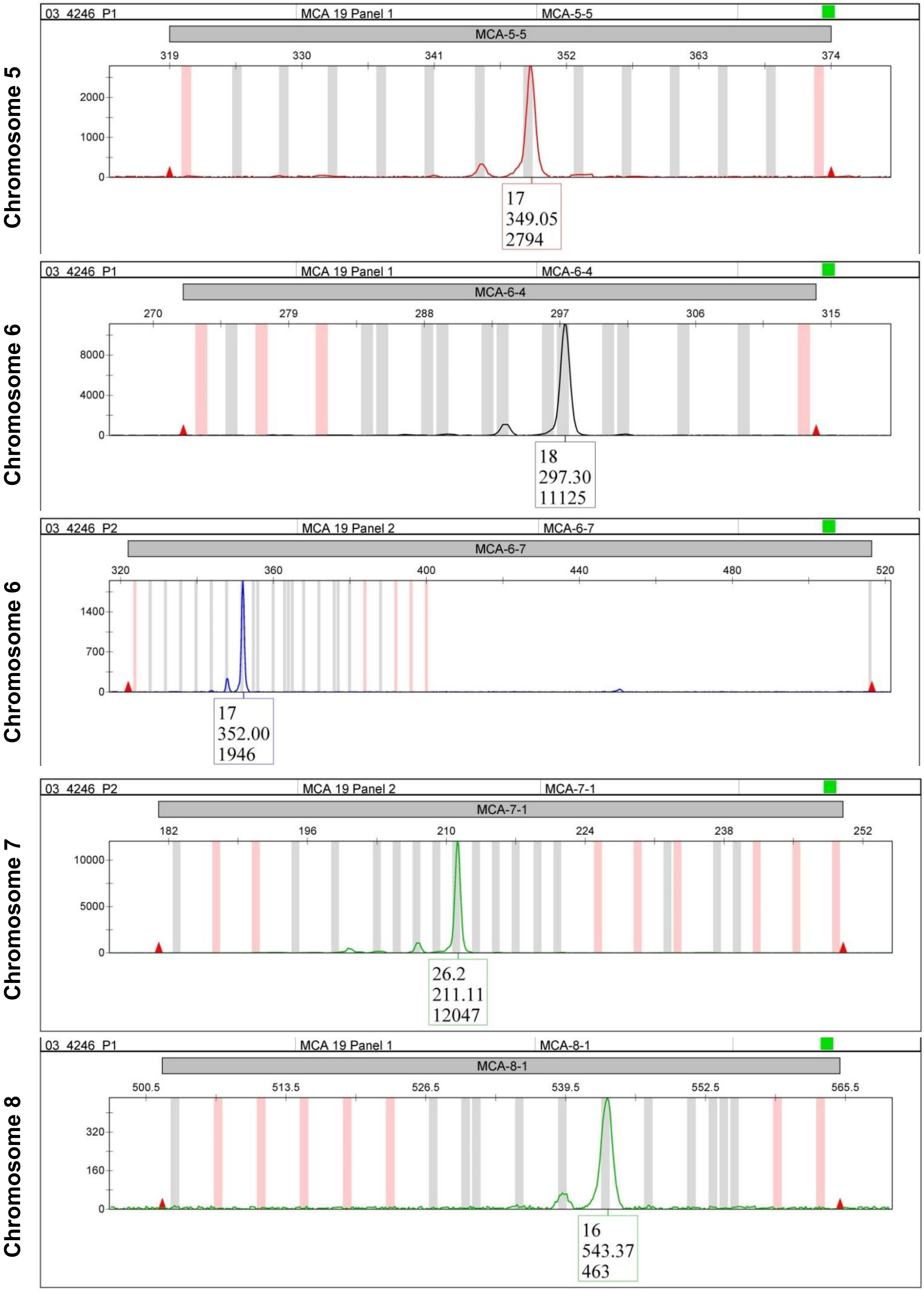
Chromosome 5
03_4246_P1
MCA 19 Panel 1
MCA-5-5
MCA-5-5
319
330
341
352
363
374
2000
1000
0
17
349.05
2794
Chromosome 6
03_4246_P1
MCA 19 Panel 1
MCA-6-4
MCA-6-4
270
279
288
297
306
315
8000
4000
0
18
297.30
11125
Chromosome 6
03_4246_P2
MCA 19 Panel 2
MCA-6-7
MCA-6-7
320
360
400
440
480
520
1400
700
0
17
352.00
1946
Chromosome 7
03_4246_P2
MCA 19 Panel 2
MCA-7-1
MCA-7-1
182
196
210
224
238
252
10000
5000
0
26.2
211.11
12047
Chromosome 8
03_4246_P1
MCA 19 Panel 1
MCA-8-1
MCA-8-1
500.5
513.5
526.5
539.5
552.5
565.5
320
160
0
16
543.37
463

Chromosome 9

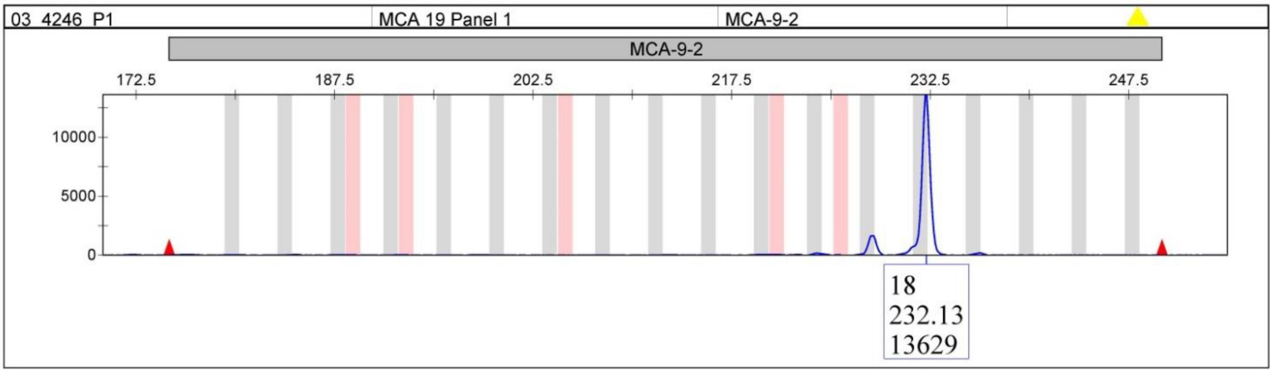

Chromosome 11

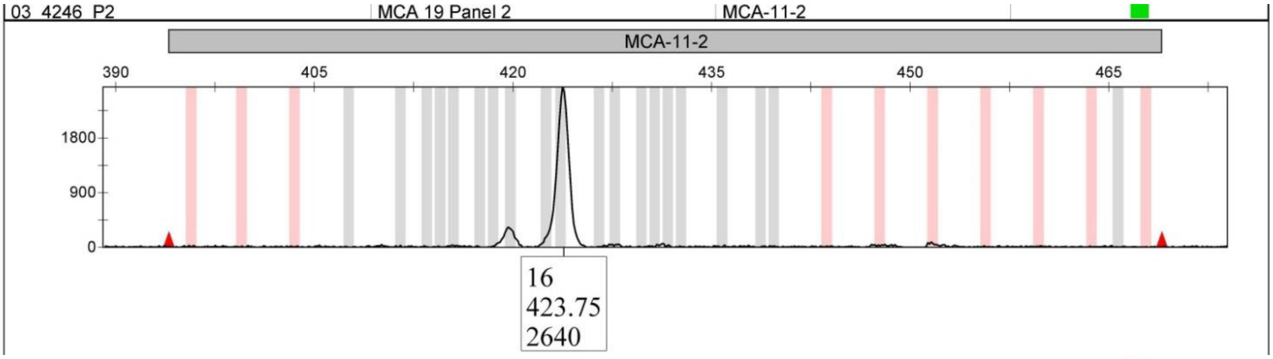

Chromosome 12

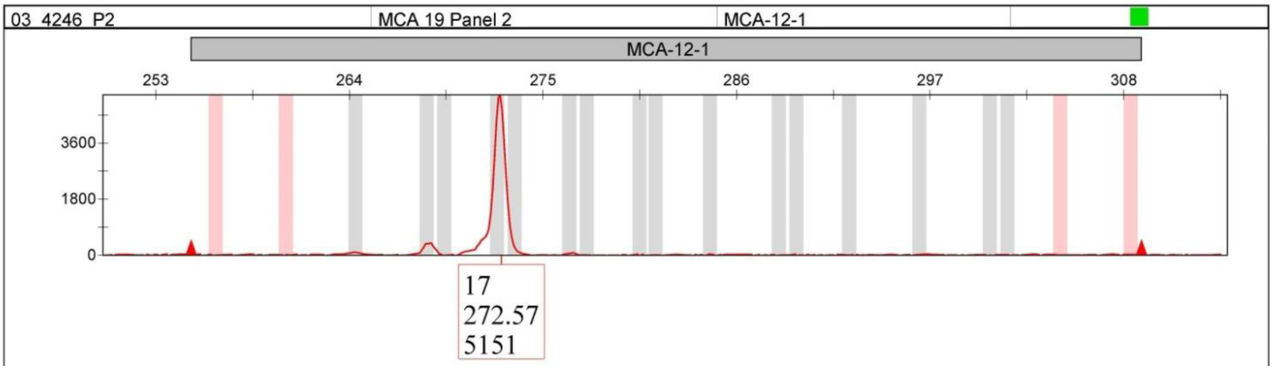

Chromosome 13

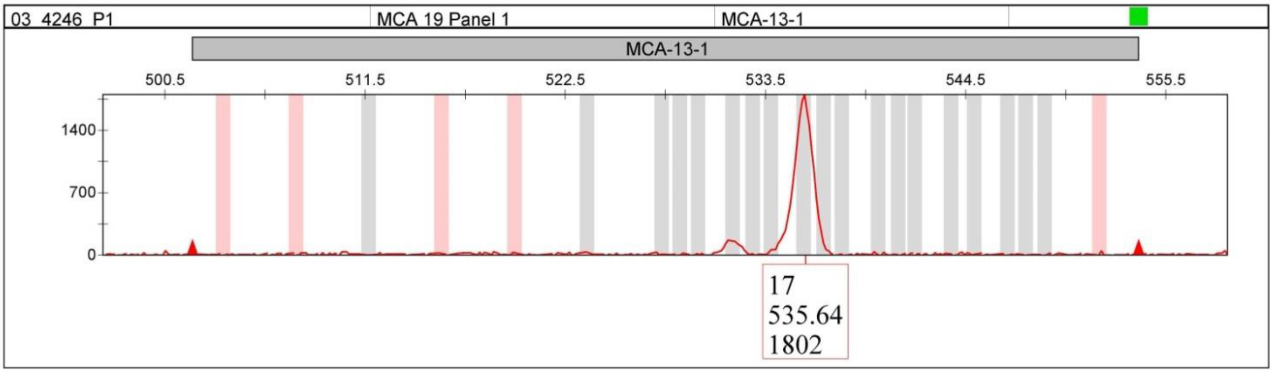

Chromosome 15

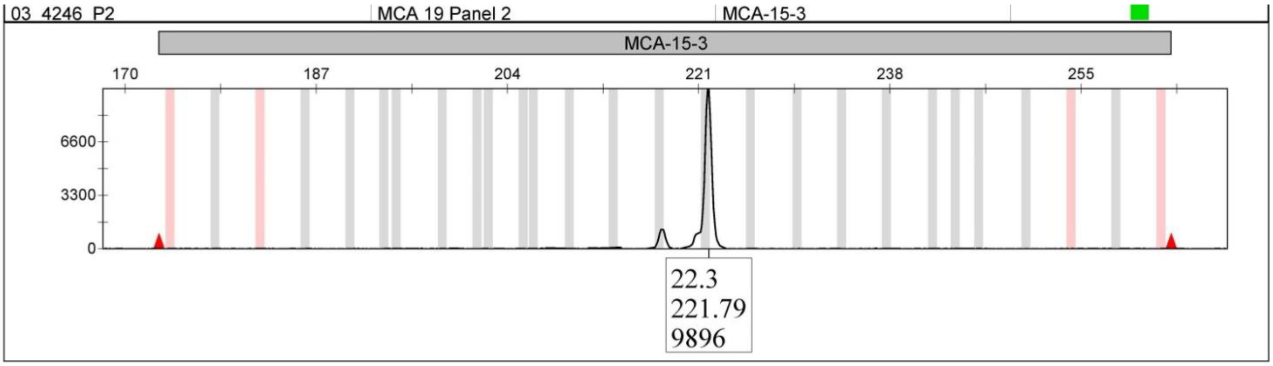

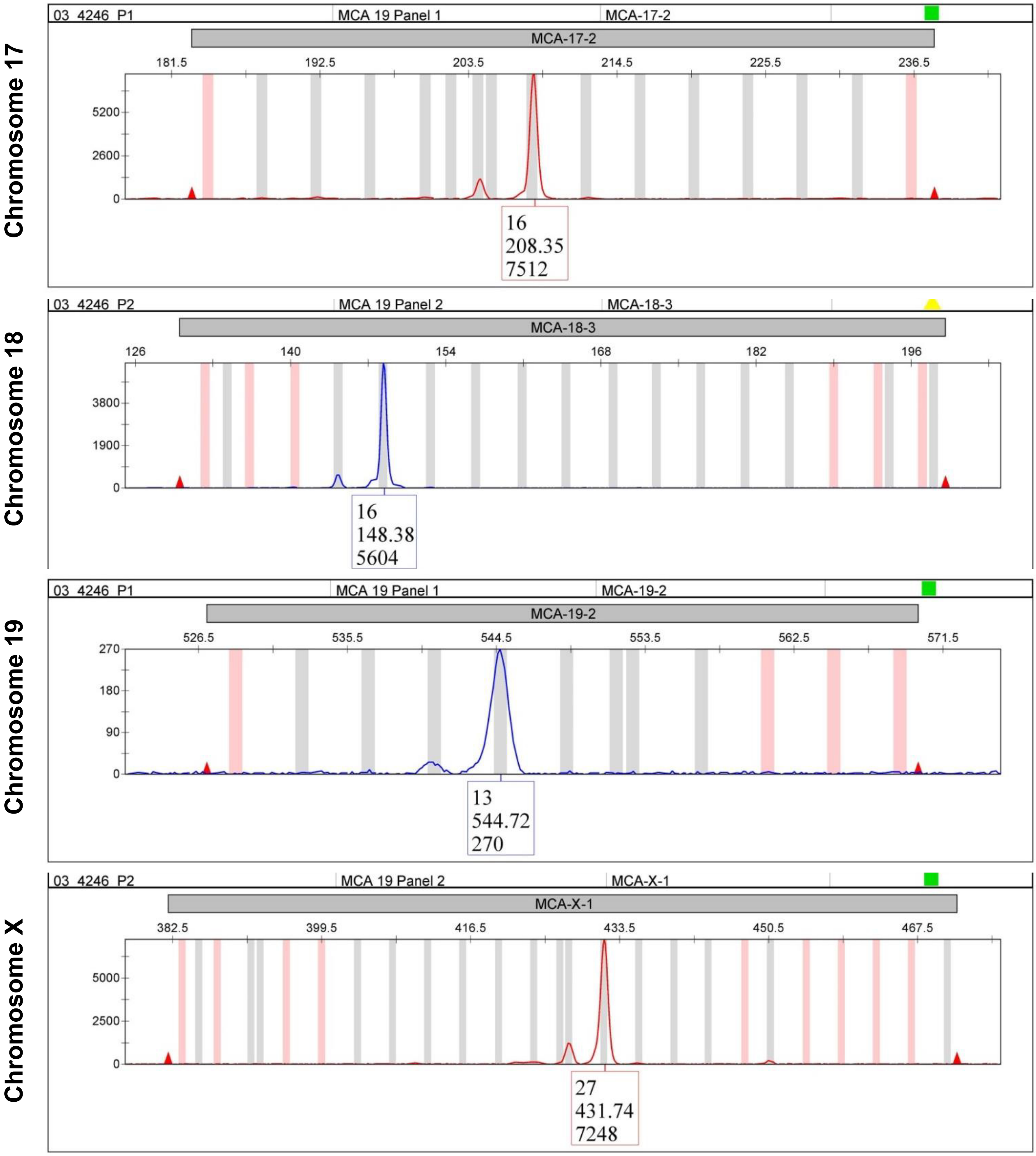
Chromosome 17
03 4246 P1
MCA 19 Panel 1
MCA-17-2
16
208.35
7512
Chromosome 18
03 4246 P2
MCA 19 Panel 2
MCA-18-3
16
148.38
5604
Chromosome 19
03 4246 P1
MCA 19 Panel 1
MCA-19-2
13
544.72
270
Chromosome X
03 4246 P2
MCA 19 Panel 2
MCA-X-1
27
431.74
7248

PAV-1
