# Supplementary material for: Genetic Characterization of Rat Hepatic Stellate Cell Line PAV-1
Source: Cells. 2023 Jun 11;12(12):1603. doi: 10.3390/cells12121603 (PMC10297474; doi:10.3390/cells12121603)
Supplement: Supplementary file 1 [file cells-12-01603-s001.zip › Table S1.pdf]

Table S1: Short tandem repeat (STR) marker analysis for AML12 cells

STR Marker	Sample Results (AML12)	CVCL_0120 (3T3-Swiss albino)	CVCL_B6Z5 (MFC/HL-041)	CVCL_0321 (HT22)	CVCL_3420 (STO)	CVCL_VR92 (MEC1)	CVCL_ZL25 (GN11)
Mouse STR 1-1 (MCA-1-1)	11	10,15	NN	10,11	12	**11**	**10**
Mouse STR 1-2 (MCA-1-2)	13	13	NN	13,17	16	13	13
Mouse STR 2-1 (MCA-2-1)	9	9	NN	9	9	9	9
Mouse STR 3-2 (MCA-3-2)	12	14	NN	14,15	14	12,13,15	14
Mouse STR 4-2 (MCA-4-2)	20.3	19.3	19.3	18.3,19.3,20.3	20.3	19.3	19.3
Mouse STR 5-5 (MCA-5-5)	14,15	13,15	13	13,14,15	14	13,14	14
Mouse STR 6-4 (MCA-6-4)	15.3	15.3	14.3	15.3	15.3	18.3	15.3
Mouse STR 6-7 (MCA-6-7)	12	12,15	12	12	12	12,15	12
Mouse STR 7-1 (MCA-7-1)	29	25.2, 29	NN	29	25.2	29	28
Mouse STR 8-1 (MCA-8-1)	14,15	15,16	NN	15	16	16,17	15
Mouse STR 9-2 (MCA-9-2)*	15	NN	NN	NN			
Mouse STR 11-2 (MCA-11-2)	18	15	NN	15,17,18	15	15,18	15,16
Mouse STR 12-1 (MCA-12-1)	19	19	19	20	19	17,18	20
Mouse STR 13-1 (MCA-13-1)	15	15	NN	16.2	16.2	15	15.2
Mouse STR 15-3 (MCA-15-3)	21.3	20.3	20.3	20.3	20.3	20.3,21.3	20.3
Mouse STR 17-2 (MCA-17-2)	13,15	12,15	NN	12,13,14	13	13	15
Mouse STR 18-3 (MCA-18-3)	21	17,21	17,21	17,19	19	17,20	17
Mouse STR 19-2 (MCA-19-2)	13	12	NN	11,12	12	12,14	12
Mouse STR X-1 (MCA-X-1)	26	26,27	26	25	26	25	26
**Identity to AML12 (%)**	**NA**	**46.81**	**44.44**	**42.31**	**41.03**	**40.82**	40.00

The search was conducted with the Cellosaurus STR Similarity Search Tool CLASTR 1.4.4 (https://web.expasy.org/cellosaurus-str-search/) containing 83 mouse cell lines with STR profile (16.11.2022). The five highest homologies found were CVCL_0120 (3T-Swiss albino, 46.81%), CVCL_B6Z5 (MFC/HL-041, 44.44%), CVCL_0321 (HT22, 42.31%), CVCL_3420 (STO, 41.03%), CVCL_VR92 (MEC1, 40.82), and CVCL_ZL25 (GN11, 40.00%) when using the following settings: Algorithm: Tanabe, Mode: Non-empty markers, Score filter: 40%, and Min. Markers: 8. * this marker is not included in the Cellosaurus STR Similarity Search Tool routine.
